# Use of Oak and Cherry Wood Chips during Alcoholic Fermentation and the Maturation Process of Rosé Wines: Impact on Phenolic Composition and Sensory Profile

**DOI:** 10.3390/molecules25051236

**Published:** 2020-03-09

**Authors:** Inês Nunes, Ana C. Correia, António M. Jordão, Jorge M. Ricardo-da-Silva

**Affiliations:** 1 LEAF—Linking Landscape, Environment, Agriculture and Food, Instituto Superior de Agronomia, Universidade de Lisboa, 1349-017 Lisboa, Portugal; inesfsnunes@gmail.com (I.N.); jricardosil@isa.ulisboa.pt (J.M.R.-d.-S.); 2 Department of Food Industries, Agrarian Higher School, Polytechnic Institute of Viseu, 3500-606 Viseu, Portugal; anacorreia@esav.ipv.pt; 3 Food and Wine Chemistry Lab, Chemistry Research Centre (CQ-VR), 5000-801 Vila Real, Portugal

**Keywords:** alcoholic fermentation, maturation process, phenolic compounds, rosé wines, sensory profile, wood chips

## Abstract

There is a lack of knowledge about the use of different wood species on rosé wine production. Thus, this work focused on the impact of the addition of wood chips from oak and cherry trees during the alcoholic fermentation and maturation process on rosé wine characteristics. Therefore, phenolic composition and sensory characteristics were monitored during the rosé wines’ production. The use of wood chips during alcoholic fermentation induced a significant increase of phenolic content in rosé musts. During rosé wine maturation, the wood chip contact induced significantly higher values of colored anthocyanins, color intensity, and polymeric pigments, and significantly lower values of color hue in the corresponding rosé wines. In terms of sensory profile, a tendency for lower scores of “overall appreciation” were attributed to control rosé wine, while significantly higher scores for “color intensity” descriptor were attributed to all rosé wines matured in contact with wood chips. For the majority of phenolic parameters and individual phenolic compounds quantified, a clear and specific influence of the use of oak and cherry wood chips was not detected, except for (+)-catechin, where the rosé wines produced in contact with cherry chips showed the highest values. This study provides relevant information for winemakers about the impact of the use of wood chips on rosé wine quality.

## 1. Introduction

In general, for rosé wine production, there are basically 3 winemaking processes: direct pressing (where red grapes are pressed without a previous red skin contact), pre-fermentative maceration (process where there is a slight contact with grape skins before fermentation), and drawing-off or *saignée* (where the grapes are crushed, stemmed, and placed in a closed vat at low temperature, following which the grape must is separated by drawing-off from the vat). In addition, the use of carbonic maceration is another option for rosé wine production [1,2,3,4,5,6].

Oenological research about rosé wines has been investigating winemaking techniques, particularly for the maceration process [3,6,7,8] and alcoholic fermentation [9], and their impact on phenolic content, aromatic composition, and sensorial profile [10,11,12]. In addition, other studies have reported on rosé wine evolution during storage [13,14,15,16]. However, it is important to note that the published works on rosé wines have not focused on the use of different wood chip species (particularly oak and cherry) during the entire wine production process (i.e., during alcoholic fermentation and the aging process). In fact, several studies have been carried out on the impact of the use of different wood species, namely oak, acacia, and cherry, on chemical and sensorial characteristics of aging of red [17,18,19,20,21,22] and white wines [23,24,25]. Recently, Santos et al. [15] reported the use of different wood chip species (acacia, cherry, and oak) in rosé wines during a very short period of aging. However, these authors only evaluated the impact of the use of several wood chip species on a short maturation time and did not evaluate the use of chips in a combined way (i.e., during the alcoholic fermentation and maturation process). Moreover, wood chip addition during alcoholic fermentation has been only studied for red and white wines [26,27] and not for rosé wines.

Thus, in order to deepen the knowledge of the impact of the use of wood chips on chemical and sensorial characteristics of rosé wines, the work reported here evaluated the phenolic composition and sensory profile of these wines during the alcoholic fermentation, with a maturation period of 80 days, in contact with chips from oak and cherry.

## 2. Results

### 2.1. Evolution of General Phenolic Composition and Color Parameters during Alcoholic Fermentation

Table 1 reports data related to the evolution of several phenolic and color parameters during the alcoholic fermentation of rosé musts in contact with oak and cherry wood chips. Thus, regarding total phenols, non-flavonoid phenols, and flavonoid phenols during alcoholic fermentation, an increment of the values of these parameters for the rosé musts fermented in contact with wood chips compared to the values quantified for the control rosé must was detected. For the total phenols index, particularly in the last 12 days of fermentation, rosé musts fermented in contact with wood chips showed significantly higher values. This tendency was also detected, in general, for flavonoid and non-flavonoid phenol compounds. However, a clear differentiation between rosé musts fermented in contact with oak or cherry wood chips was not evident.

For total anthocyanin content, a decrease in both rosé musts fermented in contact with both wood chip species from day 2 of alcoholic fermentation until the last sampling day was observed. A similar trend was also observed for the control wine. However, the decrease detected for control wine was less evident and generally showed higher values for total anthocyanins than rosé musts fermented in contact with wood chips.

For colored anthocyanins (i.e., the content of free red-colored anthocyanins in flavylium cation form), although some oscillations were detected during the alcoholic fermentation, a similar tendency to that of total anthocyanins was found (Table 1). Additionally, at the end of alcoholic fermentation, significantly higher concentrations of colored anthocyanins were detected in control must, followed by the must fermented in contact with oak chips, and finally the must fermented in contact with cherry chips.

Regarding the ionization degree of anthocyanins, rosé musts fermented in contact with wood chips showed a tendency for an increase of the values during the first 2 days of fermentation, followed by a variation of the values during all remaining fermentative processes. For control rosé must, a tendency for a variation of the values was also detected, however across the entire fermentation period. In general, through the analysis of the data shown in Table 1, it was clear that during all alcoholic fermentation, significantly higher values of ionization degree of anthocyanins were detected in rosé musts fermented in contact with both wood chip species used.

In terms of total pigments, this parameter showed a tendency to decrease throughout alcoholic fermentation, which was particularly evident for the rosé musts fermented in contact with wood chips (Table 1). In the last 12 days of fermentation, besides a continuous decrease of the values found, control rosé must showed significantly higher values compared to rosé musts fermented in contact with the two wood chip species used. For polymeric pigments and the polymerization degree of pigments, the results obtained showed a tendency for an increase of these two parameters in all rosé musts. However, this increase was more pronounced for the rosé must fermented in contact with cherry wood chips, which exhibited the highest concentrations of polymeric pigments from the sixth fermentation day onwards.

Finally, for color parameters, and specifically for color intensity, the results showed a tendency in all rosé musts for an increase of the values during the first 2 days of fermentation, followed by a slight decrease with some oscillation of the values (Table 1). After 20 days of fermentation, control rosé must showed significantly higher values for color intensity, followed by the rosé musts fermented in contact with cherry and oak wood chips (1.01, 0.87, and 0.90 absorbance units, respectively). For color hue results, a tendency for a progressive increase was detected throughout alcoholic fermentation in all rosé musts. However, this increase was more evident for the rosé musts fermented in contact with wood chips, particularly from the eighth fermentation day onwards. At the end of alcoholic fermentation, rosé must fermented in contact with cherry chips displayed significantly highest values for color hue, followed by rosé must fermented in contact with oak chips and control rosé must. During alcoholic fermentation, color due to copigmentation showed a tendency to decrease in all rosé musts. However, this decrease was more evident for rosé musts fermented in contact with wood chips.

### 2.2. Physicochemical Composition of Rosé Wines

The general physicochemical composition of the 3 rosé wines produced showed acceptable physicochemical standards. Thus, taking into account the average values for the general physicochemical characterization, the wines showed low volatile acidity (0.27 g/L acetic acid) and pH = 3.04, and also an adequate SO_2_ total and free values (151 and 38 mg/L, respectively). In addition, the rosé wines produced also showed a total acidity of 6.82 g/L tartaric acid and an alcohol degree of 13.2% (*v*/*v*, 20 °C). It was, therefore, with these initial physicochemical characteristics that the rosé wines produced were submitted to a maturation process over 80 days. It is important to note that the values obtained for these general physicochemical parameters were similar between all rosé wines, with no differences due to the type of wood chips added during the winemaking process. Then, after 40, 60, and 80 days of maturation, the wines showed average values of volatile acidity of 0.27, 0.28, and 0.30 mg/L acetic acid, respectively, while the values of free SO_2_ remained between 35 and 38 mg/L during the studied maturation period. For the pH values, these were kept between 3.04 and 3.07.

### 2.3. Evolution of General Phenolic Composition and Color Parameters of Rosé Wines During Maturation

After the production of the 3 rosé wines, they were divided following the experimental conditions described in Material and Methods. Thus, the results for the general phenolic composition and color parameters for the rosé wines matured over 80 days are shown in Table 2.

Regardless of the total phenols index, a slight decrease of the values during the maturation time was detected for the control rosé wine. For the remaining rosé wines that had contact with the different wood chip species (during alcoholic fermentation only or during the whole process), although there was some variation, the values remained stable throughout the studied maturation time. Rosé wine produced in contact with cherry chips only during alcoholic fermentation (CHF sample) and the rosé wine produced in contact with cherry chips during alcoholic fermentation and the maturation process (CHFM sample) showed the highest values for the total phenols index. The lowest values for the total phenols index were obtained for the rosé wines with oak chip contact only during alcoholic fermentation or during all maturation processes (OKF and OKFM samples, respectively). Control rosé wine showed intermediate values during the entire maturation time.

With regard to the flavonoid and non-flavonoid phenols, in general, although there were some slight variations, the values remained stable over the 80 maturation days for all rosé wines. Control rosé wine and the rosé wines obtained with cherry chip contact only during alcoholic fermentation or during the whole process (CHF and CHFM samples, respectively) generally showed the highest values for these two phenolic compounds groups, especially after 80 maturation days.

For total anthocyanins, during the entire maturation period considered, their content remained stable and similar between all rosé wines, although with some variations. In terms of quantification of the colored anthocyanins (Table 2), control rosé wine showed the lowest values during the entire maturation time (ranged from 1.72 to 2.14 mg/L malvidin-3-*O*-glucoside equivalent), while rosé wines with chip contact (only during alcoholic fermentation or throughout all processes) showed the highest values. Among rosé wines with some wood chip contact, wines obtained with cherry or oak wood chip contact during alcoholic fermentation and the maturation process showed the highest values for colored anthocyanins, particularly after 80 aging days. Additionally, for the ionization degree of anthocyanins, a similar tendency was also detected, where control rosé wine also showed the lowest values during the entire maturation time.

In terms of total pigments, a tendency for a variation of the values over maturation time was detected. However, no clear differentiation was found between the rosé wines. Nevertheless, an opposite trend was observed for the evolution of polymeric pigments (Table 2). Thus, control rosé wine showed the lowest values over the entire aging time (ranged from 0.07 to 0.08 abs. units).

Concerning the evolution of the polymerization degree of pigments, significantly low levels were detected for control rosé wine during the studied maturation time. For the remaining rosé wines, a tendency for a gradual decrease of the values was detected, without a clear differentiation between the wines. Finally, for color parameters, and specifically for color intensity, control rosé wine maintained the lowest values during the entire maturation time (values of around 0.35 abs. units), which is consistent with the same trend that had already been detected for colored anthocyanins. For the remaining rosé wines, a tendency for a slight decrease of the values was detected up to 80 maturation days (Table 2).

For color hue results, a tendency for a clear increase of the values was observed during the entire maturation time for control rosé wine. At the same time for all data points, this rosé wine showed the highest color hue values (ranging from 0.79 to 0.93 abs. units). For the remaining wines, a slight increase was detected for the rosé wines fermented only in contact with wood chips (CHF and OKF samples), while for the two rosé wines with wood chip contact during alcoholic fermentation and the maturation process (CHFM and OKFM samples), the color hue values remained almost constant (Table 2). At last, after a decrease during the alcoholic fermentation in all rosé musts, the evolution of color due to copigmentation during maturation was characterized for all rosé wines by a slight oscillation of the values during the entire studied maturation period. However, all rosé wines produced with some wood chip contact showed a slight tendency for an increment of the color due to copigmentation values, however without significant differences between them.

### 2.4. Evolution of Individual Phenolic Compounds of Rosé Wines during Maturation

**Figure 1:**
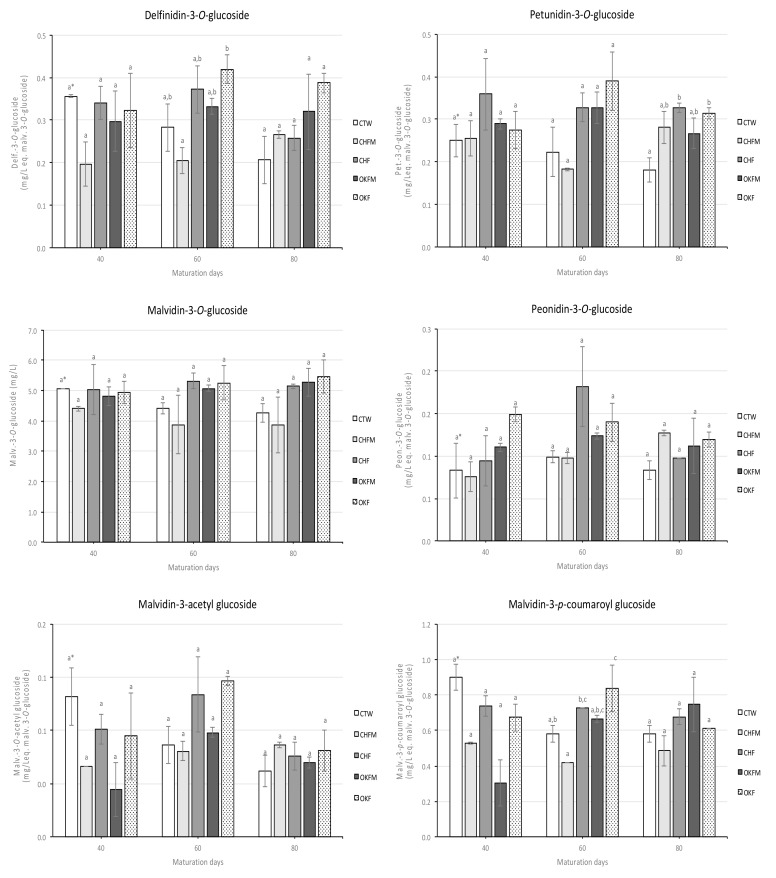
Evolution of major individual monomeric anthocyanins of rosé wines aged in contact with oak and cherry wood chips during 80 maturation days. Note: ^(1)^ for sample codes legend, see Table 3; ^*^ mean values with the same letters for each parameter and for the same maturation day are not significantly different (Tukey’s test, *p* < 0.05); average values of three replicates.

The major individual monomeric anthocyanins quantified in rosé wines over 80 maturation days are shown in Figure 1. In general, the content of individual monomeric anthocyanins oscillated over the studied maturation time for all rosé wines. In addition, the obtained results did not show a clear trend among the different rosé wines during the maturation time. Nevertheless, control rosé wine showed a tendency for lower values of the individual anthocyanins quantified; however, the differences were not substantial.

**Figure 2:**
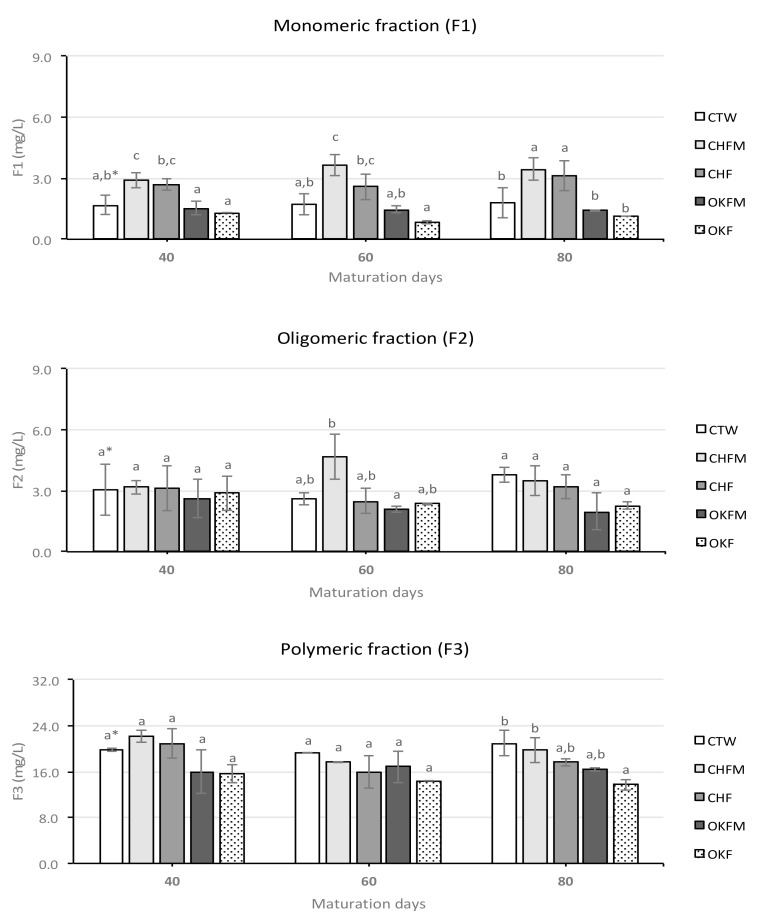
Evolution of monomeric, oligomeric, and polymeric fractions of proanthocyanidins of rosé wines aged in contact with oak and cherry wood chips during 80 maturation days. Note: ^(1)^ for sample codes legend, see Table 3; ^*^ mean values with the same letters for each parameter and for the same maturation day are not significantly different (Tukey’s test, *p* < 0.05); average values of three replicates.

The results for monomeric, oligomeric, and polymeric fractions of proanthocyanidins of the rosé wines during the maturation time are shown in Figure 2. For all rosé wines, the polymeric fractions of proanthocyanidins was the most abundant fraction during the maturation time, with the values changing between 13.73 and 22.20 mg/L. For monomeric and oligomeric fractions, similar values were found. In addition, for the three fractions of proanthocyanidins studied, their contents varied over the maturation time for all rosé wines (Table 2).

For the monomeric fraction, rosé wines produced in contact with cherry chips (CHF and CHFM samples) showed the highest values during the entire studied maturation period (ranging between 2.57 and 3.65 mg/L). On the other hand, for this proanthocyanidin fraction rosé wines produced in contact with oak wood chips (OKF and OKFM samples) showed the lowest values over the maturation time (ranging between 0.84 and 1.51 mg/L), while control rosé wine showed intermediate values (ranging between 1.67 and 1.80 mg/L). Regarding the oligomeric fractions of the proanthocyanidins, the results did not show a clear differentiation among the different rosé wines during the maturation time (except for CHF samples after 60 aging days, where significantly higher values were detected).

Finally, for the polymeric fractions of proanthocyanidins, the results did not show a clear differentiation among the different rosé wines during the maturation considered time.

**Figure 3:**
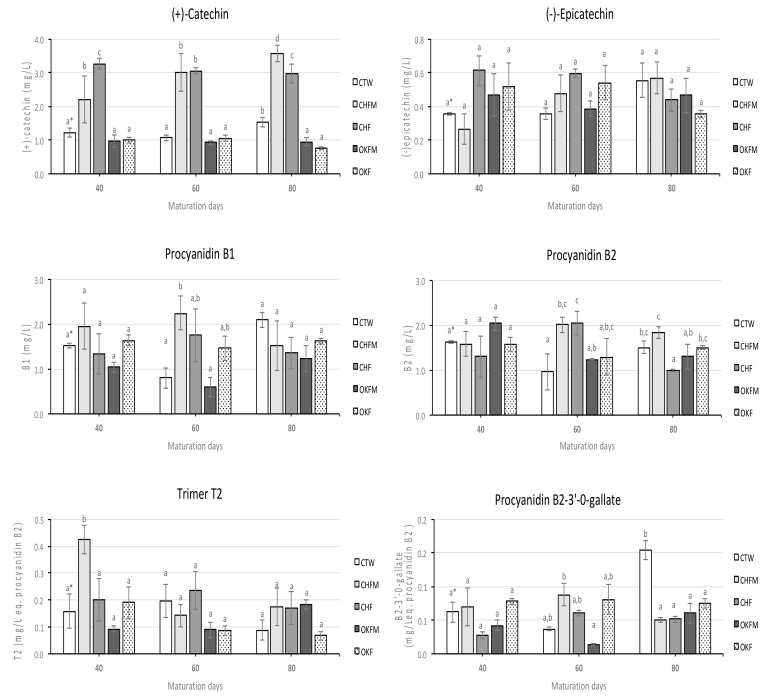
Evolution of (+)-catechin, (-)-epicatechin monomers, and several individual procyanidins of rosé wines aged in contact with oak and cherry wood chips over 80 maturation days. For sample codes legend, see Table 3; ^*^ mean values with the same letters for each parameter and for the same maturation day are not significantly different (Tukey’s test, *p* < 0.05); average values of three replicates.

Figure 3 shows the evolution of (+)-catechin, (-)-epicatechin, trimer T2, procyanidins B1, B2, and B2-3′-O-gallate quantified in rosé wines during the maturation period. For all of these individual flavonoid compounds, in general, the values varied during the maturation time in all rosé wines. Specifically for (+)-catechin, rosé wines with cherry chip contact (CHF and CHFM samples) showed the highest values (ranged between 2.20 and 3.56 mg/L) during the entire studied maturation time, followed by the control rosé wine (ranged between 1.06 and 1.53 mg/L) and the other rosé wines that were elaborated with oak chip contact (OKF and OKFM samples, which ranged between 0.76 and 1.05 mg/L, respectively). Concerning the evolution of (-)-epicatechin, in all rosé wines, the values varied over time, with no clear differentiation among them. Finally, for the individual procyanidins, dimeric forms B1 and B2 were the most abundant individual procyanidins detected during the entire maturation time (Figure 3). In addition, for all individual procyanidins detected, the values varied over time in all rosé wines, without clear differentiation among them. However, rosé wines fermented and matured in contact with cherry chips (CHFM sample) showed a slight tendency for higher values of procyanidin B2, particularly after 80 maturation days. An opposite trend was observed for procyanidin B2-3′-O-gallate, for which very small values were quantified in all wines, although after 80 maturation days it was the control wine (CTW sample) that showed the higher values.

### 2.5. Evolution of Sensory Profile of Rosé Wines during Maturation

**Figure 4:**
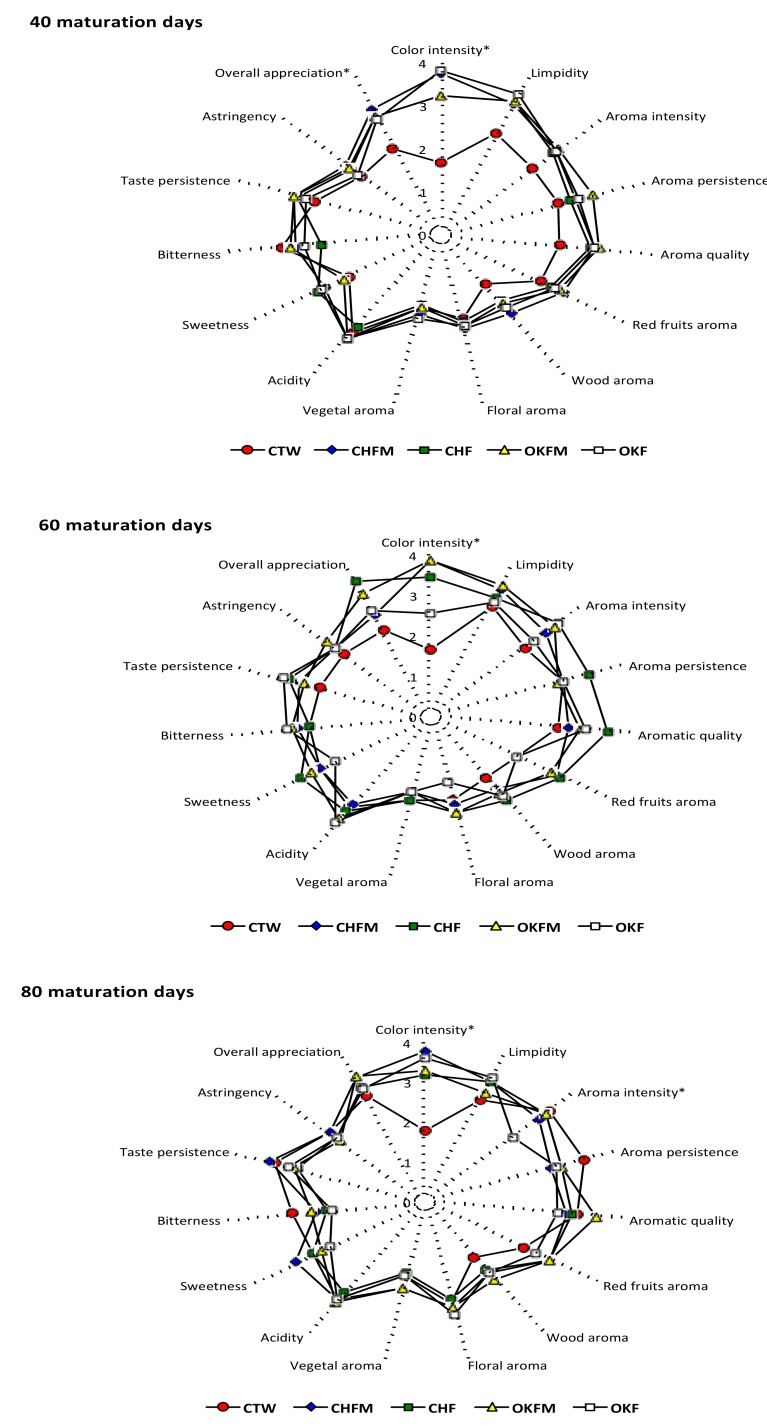
Evolution of sensory profile of rosé wines matured in contact with oak and cherry wood chips at 40, 60, and 80 maturation days. For sample codes legend, see Table 3; * sensory parameters with significantly differences between the rosé wines (Tukey’s test, *p* < 0.05).

Figure 4 shows the spider web diagrams obtained with the average values of each descriptor from sensory analysis of rosé wines during the maturation. The most marked differences during maturation of all rosé wines were related to the aroma descriptors (specially for “intensity”, “quality” and “wood”), “color intensity”, and “overall appreciation”. However, for the panel test, only “color intensity” attributes was detected as being statistically different over the entire maturation time. According to the panel test, control rosé wine after 40 maturation days showed the lowest scores for “color intensity”, “limpidity”, “sweetness”, “overall appreciation”, and also for several aromas descriptors (“intensity”, “persistence”, “quality”, and “wood”). However, only for “color intensity” and “overall appreciation” were the differences statistically different. For the remaining rosé wines, there were no significant differences between the wines. In addition, it was also clear that the differences for the majority of sensory descriptors between the rosé wines increased after 60 maturation days, particularly for the aroma descriptors, “overall appreciation”, and “color intensity”.

### 2.6. PCA Applied to Rosé Musts and Wines Characterization

To better understand the relationship between different production and maturation conditions, general phenolic parameters, individual phenolic compound groups, and sensorial attributes of rosé wines, a principal component analysis (PCA) was performed, considering three data points (40, 60, and 80 maturation days).

**Figure 5:**
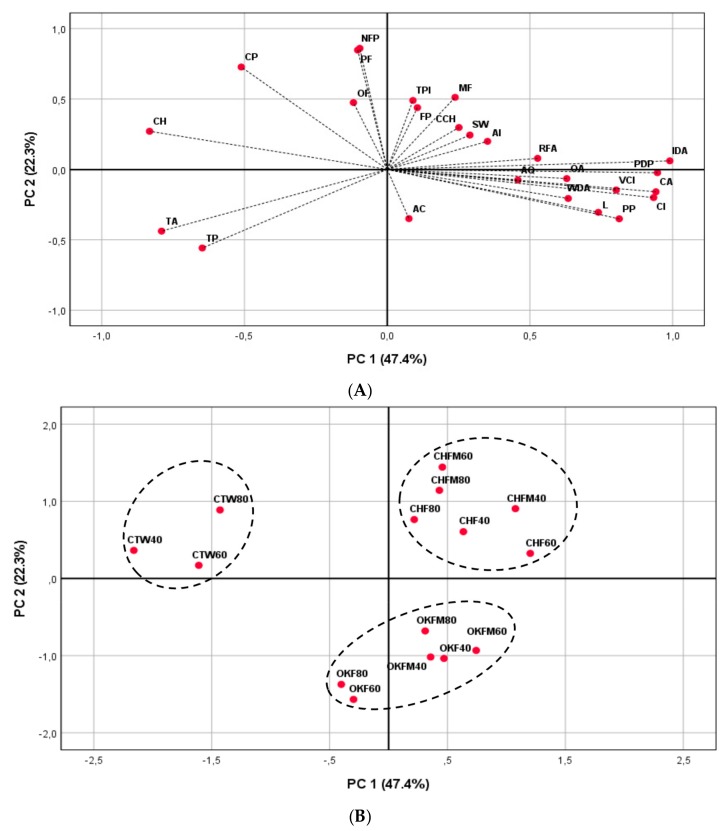
Principal component analysis (PCA; PC1 and PC2) for different sensorial attributes and several phenolic parameters of rosé wines aged in contact with oak and cherry wood chips over 80 maturation days. (**A**) Projection of sensorial attributes and phenolic parameters. (**B**) Projection of rosé wines samples. Phenolic parameters: CI—color intensity; CH—color hue; IDA—ionization degree of anthocyanins; TA—total anthocyanins; CA—colored anthocyanins; TPI—total phenols index; FP—flavonoid phenols; NFP—non flavonoid phenols; TP—total pigments; PP—polymeric pigments; PDP—polymerization degree of pigments; CP—color due to copigmentation; MF—monomeric fraction of proanthocyanidins; OF—oligomeric fraction of proanthocyanidins; PF—polymeric fraction of proanthocyanidins; CCH—(+)-catechin. Sensorial attributes: VCI—color intensity; L—limpidity; AI—aroma intensity; AQ—aroma quality; RFA—red fruits aroma; WDA—wood aroma; AC—acidity; SW—sweetness; AO—overall appreciation. CTW—control wine; OKF—wine produced with oak wood chip contact only during alcoholic fermentation; OKFM—wine produced with oak wood chip contact during alcoholic fermentation and maturation process; CHF—wine produced with cherry wood chip contact only during alcoholic fermentation; CHFM—wine produced with cherry wood chip contact during alcoholic fermentation and maturation process. Maturation periods: 40, 60, and 80 days.

The corresponding loading plots that established the relative importance of each variable are shown in Figure 5. The PCA showed that the first two PCs explained 69.7% of the total variance (Figure 5A). The first principal component (PC) (PC1, 47.4% of the variance) was positively correlated with several phenolic parameters (color intensity, ionization degree of anthocyanins, colored anthocyanins, polymeric pigments, and polymerization degree of pigments) and sensorial descriptors (“color intensity”, “limpidity”, “aroma intensity”, “aroma quality”, “red fruits aroma”, “wood aroma”, “sweetness”, and “overall appreciation”), and negatively correlated with other several phenolic parameters (color hue, color due to copigmentation, total anthocyanins, and total pigments). The second PC (PC2, 22.3% of the variance) was positively correlated with the following phenolic parameters and compounds: total phenols index, flavonoid phenols, non-flavonoid phenols, different fractions of proanthocyanidins (monomeric, oligomeric, and polymeric), and (+)-catechins. On the other hand, this second PC was negatively correlated with one taste sensation (“acidity”).

In Figure 5B, it is possible to visualize the spatial distribution of the rosé wines produced in contact with cherry or oak wood chips in regard to the different parameters considered. Thus, after a cluster analysis, three different groups were formed. One group contained all control rosé wines during the entire maturation time considered (CTW40, CTW60, and CTW80 wine samples); these wines were positively related with color hue, color due to pigmentation, total anthocyanins, and total pigments. Another group was formed from the rosé wines produced in contact with oak wood chips (OKF40, OKF60, OKF80, OKFM40, OKFM60, and OKFM80 wine samples). These rosé wines were positively related with only one sensorial taste descriptor, “acidity”. Finally, a last group was formed from all rosé wines produced in contact with cherry wood chips (CHF40, CHF60, CHF80, CHFM40, CHFM60, and CHFM80 wine samples). All of these rosé wines were positively related with some sensorial descriptors, such as “sweetness”, “aroma intensity”, and “red fruit aromas”, and also with some phenolic parameters and compounds (total phenols index, flavonoid phenols, monomeric fractions of proanthocyanidins, and (+)-catechins).

## 3. Discussion

Authors should discuss the results and how they can be interpreted in light of previous studies and of the working hypotheses. The findings and their implications should be discussed in the broadest context possible. Future research directions may also be highlighted. Overall, regarding the works published related to the effects of the use of different wood chip species (in particular oak wood) on wine composition and sensory profile, these findings have been related to red and white wines. Only recently have a few studies been published relating to rosé wines, however these were using a very short maturation period and did not consider the use of wood chips during the alcoholic fermentation process itself.

Thus, it is difficult to perform a comparative analysis with previous works, particularly taking into account the different wood species, chip concentrations, contact times, and the moment of wood chip addition.

### 3.1. General Phenolic Composition and Color Parameter Changes in Rosé Musts and Wines

Several modifications of rosé musts and respective wines were observed for the diverse color and phenolic parameters during the alcoholic fermentation and maturation process (Table 1 and Table 2). The results concerning the effects of the two wood chip species used during the alcoholic fermentation on the evolution of global phenolic parameters of rosé musts showed significant changes in the majority of the parameters studied, however, without clear and specific differentiation according to the species of wood used. Thus, concerning the potential impact of the two wood chip species used, the results obtained demonstrated that the use of oak and cherry woods generally induced a higher increase of phenolic content of rosé musts. The high total phenols index, flavonoid content, and non-flavonoid content of rosé musts fermented in contact with wood chips compared to control rosé must could correspond to a higher extraction of several individual phenolic compounds, such as (+)-catechin, gallic acid, ellagitannins, and ellagic acid, during alcoholic fermentation. In addition, from the dynamics detected specifically for the flavonoid phenols evolution, it seems that only after more than one week of alcoholic fermentation (after 10 days) was it possible to detect an increase of the contents of these compounds groups (Table 1). It will likely be necessary to first allow time for the grape must impregnation into the wood chips to induce an extraction of these compounds during the alcoholic fermentation. At the same time, if the musts have a high alcohol content (particularly at the end of alcoholic fermentation) will a remarkable increase of these compounds be possible, because alcohol can help to increase the solubility of the wood compounds into the musts. For some authors, phenolic compound extraction from wood to wine will be dependent on the level of wine penetration into the wood, the concentration gradient between wine and wood, and also the natural phenolic richness of the wood species [28].

In general, the tendency that was verified for the mentioned global phenolic parameters during alcoholic fermentation was maintained during the entire aging time (Table 2). Thus, values remained stable, and higher values were seen for wines fermented in contact with cherry wood chips and those still in contact with them during the maturation time. Del Álamo et al. [29] reported a progressive decrease of total phenols in wines during the first months of maturation in contact with chips, while after the third month an increase of the values was observed. Moreover, other authors [27,30] described an increase of total phenols as a result of wood chip addition during fermentation after two months of contact with the wine. Therefore, the results obtained in our experimental work for all rosé musts and wines confirmed the tendency previously reported by other authors for higher total phenolic content in red [17,18,19,20,21], white [23,24,25], and rosé [15] wines aged in contact with different wood chips.

The results obtained for total anthocyanins showed a tendency for a decrease of the values in all musts during alcoholic fermentation. However, this decrease was more evident for the musts fermented in contact with wood chips, independently of the two wood species used (Table 2). During the wine maturation process, although there was some variation, the values remained stable and without significant differentiation between rosé wines (Table 2). A similar tendency was also observed for colored anthocyanins during the alcoholic fermentation, in particular after the eighth day of fermentation, where there was an evident decrease of colored anthocyanins in musts fermented in contact with wood chips. In addition, for the ionization degree of anthocyanins, a similar tendency was detected, although this decrease was generally gradual and slight in all musts. These findings could derive from oxidation reactions during alcoholic fermentation or from condensation reactions between anthocyanins, condensed tannins, and also different wood molecules, all of which would generate insoluble and precipitable polymers. During rosé wine maturation, there was a general decrease in colored anthocyanins values, although the wines fermented and matured in contact with wood chips always maintained the highest values and at same time had a less marked decrease of the values compared to control rosé wine. A similar tendency was also detected for the ionization degree of anthocyanins during the maturation process. This parameter represents the percentage of anthocyanins in the flavylium cation form at the pH of wine. Of course, all of this evolution was reflected in the evolution of color intensity during fermentation, and in particular during rosé wine maturation (Table 1 and Table 2).

Thus, significantly higher values for color intensity were clearly detectable in rosé wines that had contact with the wood chips only during alcoholic fermentation or those that maintained the wood chip contact during the maturation process compared to control rosé wine. However, there was no clear differentiation when considering the wood chip species used. It is also important to note that these differences detected between wood chip contact rosé wines and control rosé wine are consistent with the results obtained for the “color intensity” sensorial descriptor, as can be seen from the sensory profile results showed in Figure 4.

The results obtained in our work for rosé wine maturation are, therefore, generally in agreement with previous published works for red wines [18,21,27]. In fact, the use of wood promotes pigment stabilization, namely of anthocyanin pigments, and induces a higher color intensity and the best chromatic attributes in wines. However, the published works show some contradictions because other authors [22] described a continuous decrease in the anthocyanin contents for red wines aged in contact with wood during 12 months, thus provoking a reduction of red color. These findings could derive from oxidation reactions during aging or from condensation reactions between anthocyanins and certain wood molecules, all of which would generate large, insoluble, and precipitable polymers. In addition, Santos et al. [15] reported no differences for color intensity between rosé wines aged in contact with different wood chip species over 20 aging days. Furthermore, several authors point out the aging time as the main factor affecting the physicochemical features of wines aged in contact with wood [19,29].

Our results also demonstrated that rosé musts fermented in contact with wood chips showed the highest values for color hue, particularly from the eighth day of fermentation, and with greater incidence for the must in contact with cherry wood chips. However, wood chip contact during alcoholic fermentation, and in particular the maintenance of their contact during the wine maturation, induced a positive effect through significantly lower color hue values detected in rosé wines. In the opposite way, control rosé wine showed the highest values for color hue across the entire maturation period. It is important to emphasize that this high color hue is a result of an increase of yellow and brown components, due to the oxidation reactions involving the different wine phenolic compounds during the maturation process [22,29]. Thus, the results obtained in our work prove the positive effect of the rosé wine’s contact with the two wood chip species used, allowing the compounds extracted from the wood to help in color stabilization and protection, due to the formation of more stable polymeric complexes. According to results reported by Jordão et al. [31], the presence of ellagic tannins extracted from oak wood also increase the protection of wine phenolic components against the oxidation process. In addition, recently Santos et al. [15] described the lowest values for color hue for rosé wines matured over 20 aging days in contact with cherry wood chips. Although the concentration of wood chips used has been low, the wood chip contact during only alcoholic fermentation, as well as their maintenance during the maturation, seems to have a positive influence on the colored anthocyanin content, ionization degree of anthocyanins, and also on the color intensity and hue of the rosé wines. However, it was not possible to detect a specific influence of the two wood chip species used. Nevertheless, according to Kyrealou et al. [27], the addition of wood chips during alcoholic fermentation did not favor ellagitannin extraction or the reactions involved in tannin condensation and anthocyanin stabilization. Thus, according to these authors the higher color intensity and lower color hues of red wines were acquired only when wood chip addition took place after fermentation.

The results obtained for total pigments, in general, showed a clear tendency for a decrease of the values in all rosé musts, especially for the musts fermented in contact with wood chips. During the maturation process, stabilization of total pigment values was observed, with no significant differences between the rosé wines (Table 1 and Table 2). However, the highest differences were observed for polymeric pigments and polymerization degree of pigments. Thus, for these two parameters it was possible to detect an increase of the values, particularly for the rosé must fermented in contact with cherry wood chips. These results allow us to consider that the use of cherry chips, in particular during fermentation, could induce a faster evolution of phenolic compounds and a fast increase in the formation of derived and polymeric compounds.

However, during the wine maturation, all rosé wines showed a tendency for a slight decrease of the polymeric pigment values. The results obtained in our work, especially for rosé musts, are in agreement with other authors, who reported a faster evolution of wine pigments with a fast increment of polymeric compounds in red wines aged in cherry barrels or aged in contact with cherry chips [11,21].

The color due to copigmentation is a phenomenon where anthocyanins begin to interact with other wine compounds, such as flavonoids, amino acids, organic acids, and with their own anthocyanins, to form more complex structures [32,33]. In addition, the anthocyanin and copigment concentrations promote this process. In our work, during alcoholic fermentation, color due to copigmentation decreased in rosé musts with wood chip contact, while control rosé must showed a tendency for an oscillation of the values over the time. According to Darias-Martin et al. [34], copigmentation phenomena in grape musts occur naturally. However, to our knowledge there are no data about color due to copigmentation in musts during alcoholic fermentation, and particularly for rosé musts. Nevertheless, several authors reported values that supported the contribution of copigmentation to the color for young red wines, which ranged 30%–50% [35] and 32%–43% [36]. With respect to the evolution of color due to copigmentation during rosé wine maturation observed in our work, there was a slight increase of the values, especially for rosé wines with wood chip contact. However, the higher values were detected for control rosé wine (ranging from 41.48% to 43.02%) and also for rosé wines that maintained contact with wood chips during the maturation (ranging from 36.64% to 42.90%).

### 3.2. Evolution of Individual Phenolic Compounds of Rosé Wines

Regarding individual monomeric anthocyanins, the evolution observed followed the same trend detected for total anthocyanin content (i.e., without a well-defined pattern among the several rosé wines). In addition, as expected, malvidin-3-*O*-glucoside was the most abundant individual anthocyanin quantified, which confirms previous published works for rosé wines [6,7,37]. On the other hand, malvidin-3-acetyl glucoside was the least abundant individual monomeric anthocyanin quantified. Several published works [6,36] reported that all anthocyanins increase with increasing maceration time during rosé winemaking. According to Kelebek et al. [37], the maceration time also had a significant effect on the individual anthocyanin content of rosé wines, where malvidin-3-*O*-glucoside ranged from 24.0 to 32.6 mg/L. However, the values reported by these authors were much higher than the malvidin-3-*O*-glucoside content quantified in our work (values ranged from 4.3 to 5.0 mg/L after 40 maturation days). This difference is due to the fact that in our research, rosé wines were made through a direct pressing procedure (i.e., without any maceration time).

In general, during rosé wine maturation, control wine showed a slight tendency for lower values of individual anthocyanins compared with the rosé wines produced with wood chip contact. Recently, Jordão et al. [38] reported significant lower levels of free monoglucoside anthocyanins in synthetic solutions containing different wood extracts (oak, acacia, or cherry) and grape skin anthocyanin extracts than in control solution, which contained only grape skin anthocyanin extracts. According to these authors, the mean loss of the monoglucoside anthocyanin level was around 45% in solutions containing wood extracts, indicating a drastic reduction of free anthocyanin pigments. Other authors also reported 30% lower levels of malvidin-3-*O*-glucoside after 20 days of contact with wood extracts in model wine solution [39]. This decrease is basically a consequence of reactions between anthocyanins and ellagitannins extracted from the wood [31]. However, other authors reported a positive impact of oak wood compounds in the individual protection of anthocyanins, namely against oxidation [40,41]. Despite these different trends reported by various authors, in our work it was not possible to detect clear differences for individual anthocyanins among the different rosé wines. This may be due to the fact that the rosé wines studied had a very low individual anthocyanin content, and as such it was difficult to detect clear differences between them.

For oligomeric and polymeric fractions of proanthocyanidins, the results did not show a clear differentiation among the rosé wines during maturation (Figure 2). However, for monomeric fractions of proanthocyanidins, rosé wines produced with cherry wood chip contact showed the highest values during the entire studied maturation time. This result is directly related to the values quantified for (+)-catechin in rosé wines that had contact with cherry wood chips (Figure 3). In this case, it was also these rosé wines that showed significantly higher (+)-catechin contents throughout the studied aging process. Chinnici et al. [19] reported significant changes in (+)-catechin and procyanidins B1 and B2 in red wines aged in cherry barrels. In fact, several authors found a great variety of individual flavonoid compounds in cherry woods, mainly high levels of (+)-catechin and B-type procyanidin dimer [42,43,44]. Thus, this could explain the higher values of (+)-catechin found in rosé wines that had contact with cherry chips, due to the extraction of this compound from wood to rosé wine. For procyanidin B2, the differences were not so marked, but slightly higher values were also found in rosé wines produced with cherry chip contact.

### 3.3. Sensory Profile of Rosé Wines

Despite the several changes observed for the different phenolic parameters studied, in sensorial terms, the differences between the studied rosé wines were not totally evident for the majority of the sensorial descriptors (Figure 4). The low wood chip concentration used (1.5 g/L) during the alcoholic fermentation and maturation process may have contributed to the low sensory differences detected between wines by the tasting panel. However, after 40 maturation days, a clear differentiation between control rosé wine and rosé wines produced with wood chip contact was evident for the majority of the aroma descriptors. This is a consequence of the extraction of wood components in the wines, such as β-methyl-γ-octalactone, furfural, eugenol, vanillin, and syringaldehyde. According to several authors [15,17,45], the presence of all of these compounds had an important role in several wine aroma descriptors. In addition, all of these compounds are detected in oak and cherry wood species [44,46,47]. In addition, it should also be noted that the use of the two wood chip species did not induce a clear differentiation between the rosé wines in terms of sensory profiles. In fact, the absence of significant differences between wines aged in contact with different wood species is not entirely new. Previously, Fernández de Simon et al. [20] reported the volatile composition and sensorial characterization of red wines aged in wood barrels from different species, including oak and cherry, and concluded that wines aged in oak were the best valuated during the entire aging time, but the differences were not always significant.

Several authors have reported on the addition of wood chips during and after alcoholic fermentation, particularly from oak species in red and white wines [26,27,48,49]. In general, for some of these authors the variable with the greatest effect on the sensory profile of the wines was the amount of wood chips used, which ranged from 2.5 to 7.0 g/L (substantially higher values than those used in this work). In addition, according to the results reported by Rodriguez-Bencomo et al. [50] for Tempranillo wines, the presence of oak chips during alcoholic fermentation enhanced the formation of ethyl esters and fusel alcohol acetates, which contributed to a reduction of lactones and volatile phenols in wines. This fact contributed to less wine differentiation in terms of sensory profile, particularly for aroma descriptors. In addition, the aromas in wines originating in the grapes and in fermentation could interact with wood components, inducing a decrease of the impact of wood chips on wine sensorial characteristics.

According to the results obtained, it was also clear that under our experimental conditions, the use of wood chips did not determine an increment of the wine astringency and bitterness perception by the tasters, although the rosé wines produced with cherry chip contact showed the highest values of (+)-catechin. This could be explained by the low wood chip concentration used in this work, combined with the low values of (+)-catechin quantified. In addition, the oxidation and precipitation of the phenolic compounds during the maturation process may help us to explain the absence of differences in the astringency and bitterness between the rosé wines [51]. These last two aspects are particularly evident in wines with low phenolic content, such as rosé wines.

It was for “color intensity” descriptor that the differences between the rosé wines were most consistent. Thus, control rosé wine showed the lowest scores for the “color intensity” descriptor during the entire maturation time. This tendency follow the same trend as the results obtained for colored anthocyanins, ionization degree of anthocyanins, and color intensity values shown in Table 3, in which control rosé wine showed the lowest values. All rosé wines produced with wood chip contact showed the highest scores for the “color intensity” descriptor during the entire aging time. This result was independent of the wood chip species used. Finally taking into account the “overall appreciation”, there was clear a tendency for lower scores attributed to control rosé wine during the entire studied maturation time. This trend was particularly evident after 40 maturation days, with significantly lower scores being see for control wine compared with the other rosé wines. However, during the entire studied maturation time, it was possible to detect a tendency towards scores closer to the other rosé wines. In any case, the results of this work have clearly shown that rosé wines with some contact with both wood chip species used always have a higher preference in terms of their overall sensory evaluation.

## 4. Materials and Methods

### 4.1. Grapes and Winemaking Process

One Portuguese red grape variety, Touriga Nacional (*Vitis vinifera* L.), was harvested in 2016 in a healthy conditions from a vineyard located in the Dão region (northwest of Portugal) for the rosé wines production used in this experiment. The rosé wines were elaborated by the Casa da Passarella winery, also located in the same region, following the direct pressing winemaking procedure. Thus, there was no maceration based on the winery option, and also as a result of the high phenolic content quantified in the grapes at harvest (total phenols index of 92.3 abs. units). The sulfitation of the grapes (55 mg/L of SO_2_) was followed by the alcoholic fermentation, which was carried out in stainless steel tanks (1000 L) using a standard *Saccharomyces cerevisiae* yeast strain (Fermol Arôme Plus by AEB Group) and inoculated at 20 g/hL. The fermentation was completed in 20 days, keeping the temperature around 20 °C. After the alcoholic fermentation, the produced rosé wines were racked and removed from contact with the lees. The produced rosé wines did not undergo malolactic fermentation and no fining treatments were made.

### 4.2. Experimental Conditions

The experimental work was conducted on rosé musts and wines. Thus, for musts, 2 different assays were conducted, while for the rosé wines produced, 4 different essays were considered. Briefly, during alcoholic fermentation, two rosé musts were fermented separately in contact with wood chips (1.5 g/L) from oak (*Quercus petraea* L.) and cherry (*Prunus avium*) species in stainless steel tanks measuring 1000 L. Then, the two rosé wines produced using the experimental conditions mentioned during alcoholic fermentation were divided into 4 different trials (2 maintaining the wood chip contact and the other two without wood chip contact) and conducted in stainless steel tanks measuring 500 L, according to the experimental conditions described in Table 3. For the essays using rosé musts and wines, a control essay (without wood chip contact) was also considered in our study.

In this experiment, the wood chips from oak (*Quercus petraea*) and cherry (*Prunus avium*) were purchased by AEB Bioquímica company (Viseu, Portugal). All of the used wood chips presented a medium toasting level and a particle dimension of 8 mm (average size). In all assays (for rosé musts and wines), a concentration of 1.5 g/L of wood chips was used. This concentration of wood chips took into account previous work carried out on rosé wines, and also the low phenolic content usually quantified in these wines [15]. All musts and wine samples were filtered (pore diameters of 13 μm) before laboratory analysis.

### 4.3. General Physicochemical Characterization

The general wine physicochemical characterization (pH, total and volatile acidity, alcohol strength, total and free sulfur dioxide) was performed following the analytical methods recommended by International Organization of Vine and Wine (OIV) [52]. All analyses were done in triplicate.

### 4.4. Determination of Global Phenolic Parameters 

The total phenol index was determined according to the methodology of Ribéreau-Gayon et al. [4], while non-flavonoid and flavonoid phenol indices were determined using the methodology described by Kramling and Singleton [53]. Total pigments, total anthocyanins, degree of ionization of anthocyanins, colored anthocyanins, polymeric pigments, degree of polymerization of pigments, polymeric pigments index, and color due to copigmentation determinations were analyzed as described by Somers and Evans [54]. Color intensity at 420, 520, and 620 nm, as well as color hue, were also evaluated following the methodology described by OIV [52]. All of these analysis were done in triplicate.

### 4.5. Fractionation of Proanthocyanidins According to Their Degree of Polymerization

A method described by Sun et al. [55] was used to fractionate rosé wine proanthocyanidins according to their degree of polymerization for catechins (monomers), oligomeric fractions (degree of polymerization ranging from 2 to 12), and polymeric fractions (degree of polymerization > 12) using a C_18_ Sep-Pack column. Thus, each sample was passed through the two preconditioned neutral Sep-Pack cartridges connected in series. To eliminate phenolic acids, a 4-mL dealcoholized medium was adjusted to a pH of 7.0 and then passed through the two connected Sep-Pack cartridges, which were preconditioned with 10 mL of water adjusted to a pH of 7.0. After drying the column with N_2_, the elutions were first carried out with 25 mL ethyl acetate to elute catechins and oligomeric proanthocyanidins, and then the polymeric fraction was eluted with 10 mL methanol. Regarding the separation of monomeric from oligomeric fractions, both fractions were completely evaporated under a vacuum at 25 °C, dissolved in distilled water, and then redeposited onto the same connected cartridges that were preconditioned with distilled water. After drying the cartridges with N_2_, catechins and oligomeric proanthocyanidins were eluted sequentially with 25 mL diethyl ether (catechins fraction), and finally with 10 mL methanol (oligomeric fraction). For each previously obtained fraction, the flavanols were quantified using the modified vanillin assay described by Sun et al. [56].

Thus, the vanillin reaction with catechin fraction was carried out in a 30 °C water bath for 15 min, and measurement of absorbance at 500 nm was at the same temperature. Finally, for oligomeric and polymeric fractions, both the vanillin reaction and measurement of absorbance at 500 nm were performed at room temperature, and the maximum absorbance was taken as the measured value. All analyses were done in triplicate.

### 4.6. HPLC Analysis of Individual Flavanols

For (+)-catechin, (-)-epicatechin, and individual procyanidins analysis, previous separation was required via the method described by Ricardo-da-Silva et al. [57]. Thus, the prior separation of individual compounds was carried out using a polyamide column for each fraction, and then each compound was identified through HPLC. Therefore, for each rosé wine sample, 15 mL were directly fractioned through sequential elutions in the polyamide column: first using 80 mL of buffer solution (pH = 7) to eliminate some compounds, (250 × 4.0 mm, particle size 5 mm) such as phenolic acids, then 50 mL of acetonitrile to elute catechins, followed by 50 mL of acetone to elute oligomeric procyanidins. After this prior separation process, rosé wine samples were then analyzed by high performance liquid chromatography (HPLC) with the use of a Merck Hitachi system (Merck, Darmstadt, Germany) equipped with a Model L-7100 pump and with a Waters 2487 dual absorbance detector. The column used (250 x 4.0 mm, particle size of 5 mm) was a RP_18_ Lichrocart^®^ 100 (Merck, Darmstadt, Germany) protected by a guard column of the same material. The HPLC conditions followed the procedure method described by Ricardo-da-Silva et al. [57]. Thus, for catechins, the solvents used were (A) acetic acid/bidistilled water (2.5:97.5 v/v) and (B) acetonitrile/solvent A (80:20 v/v), while for individual procyanidins, the solvents were (A) acetic acid/bidistilled water (10:90 v/v) and (B) bidistilled water. For catechins (which included (+)-catechin and (-)-epicatechin), the linear gradient used 93% (A) and 7% (B) for 26.1 min, followed by 88% (A) and 22% (B) for 90 s, and a “washing” period of 15 min with running methanol/water (50:50 v/v). For individual procyanidins, the linear gradient was run with the 10% (A) and 90% (B) to 70% (A) and 30% (B) for 45 min, followed by another linear step of 90% (A) and 10% (B) for 25 min, after which it remained constant for 12 min. The flow rates used were 0.9 and 1.0 mL/min, respectively, for catechins and individual procyanidins. The detection was made at 280 nm and the injection volume sample was 50 μL for both compound groups. Catechins and individual procyanidins were quantified by calibration curve, which was obtained with standard solutions of each individual compound, except for procyanidin T2 and B2-3′-O-gallate, which were expressed as equivalents of procyanidin B2. The chromatographic peaks of each compound were identified according to reference data, which was previously described by Ricardo-da-Silva et al. [57] and later confirmed by Monagas et al. [58]. All of these HPLC analyses were performed only on rosé wine samples and were done in triplicate.

### 4.7. HPLC Analysis of Individual Monomeric Anthocyanins

A Perkin Elmer HPLC system (Norwalk, Connecticut, USA) was used for analysis of major individual monomeric anthocyanins. It was equipped with a 200LC pump, connected to a ultraviolet/visible (UV/VIS) LC-95 detector and a manual injector (Rheodyne 7125-A), fitted with a 20 mL loop and a Konik absorbance monitor linked to a Konichrom data station (Konik Instruments, Barcelona, Spain). The column used was also a RP_18_ Lichrocart^®^ 100 (Merck, Darmstadt, Germany), with the same characteristics described previously for individual flavanols. The solvents were (A) 40% formic acid, (B) pure acetonitrile, and (C) double bidistilled water. The methodology used followed the conditions previously described by Dallas and Laureano [59]. Thus, the initial conditions were 25% (A), 6% (B), and 69% (C) for 15 min, followed by a linear gradient to 25% (A), 25.5% (B), and 49.5% (C) for 70 min, with a flow rate of 1 mL/min. The injection volume sample was 20 μL and the detection was made at 520 nm. Individual anthocyanins were quantified by calibration curve, which was obtained with standard solutions of malvidin-3-*O*-glucoside. The chromatographic peaks of anthocyanins were identified according to reference data previously described by Dallas and Laureano [59].

### 4.8. Sensory Evaluation

For the different rosé wines produced, a sensory evaluation over 80 aging days was carried out by eight expert judges with wine tasting experience to evaluate the rosé wines. Each rosé wine sample was stored for 24 h at room temperature before sensory analysis, which was performed at 20–22 °C in a sensory analysis room, with individual booths for each expert and according to standardized procedures [60]. Wine samples were presented to the panel in tasting glasses marked with three-digit numbers and in a randomized order. All sensory evaluation sessions were conducted in the morning from 10:00 to 12:00. Each taster evaluated five wine samples per session, over a total of three sessions, corresponding to each wine maturation time: one session after 40 maturation days, another after 60 maturation days, and the last one after 80 maturation days.

All expert judges were previously selected and trained in considering the sensorial attributes of rosé wines. During this training period, several sessions were carried out in order to train judges about the meaning of each attribute and on how to achieve intensity rating in a reliable way. All wine sensorial attributes used were selected by consensus in order to adequately describe the rosé wine appearance, aroma, and taste sensory similarities and differences under supervision of the panel leader.

The sensorial attributes used were grouped into the following way: aspect (color intensity and limpidity), aroma (intensity, persistence, quality, red fruits, wood, floral, and vegetal), taste (acidity, sweetness, and bitterness), mouthfeel sensations (persistency and astringency), and overall appreciation. The persistency attribute was considered as the ability of wine tastes and aromas to remain present in the mouth after wine had been swallowed. For each mentioned sensory attribute, the experts scored the attributes (aspect, aroma, taste, and mouthfeel sensations) on a five-point scale (1 = absence; 2 = little intensity; 3 = moderate intensity; 4 = intense; 5 = high intensity), while overall appreciation was also scored on a five-point scale (0–1 = bad; 2 = unpleasant; 3 = pleasant; 4 = good; 5 = very good).

### 4.9. Statistical Analysis

The data are presented as mean ± standard deviation. Phenolic and sensorial parameters were statistically tested by analysis of variance (ANOVA, one-way). A Tukey’s test (*p* < 0.05) was applied to the data to determine significant differences between rosé musts and wines. In addition, a principal component analysis (PCA) was also used to analyze the data and to study the relations among the rosé wines produced in contact with the different wood chip species, and their phenolic and sensory characteristics during the studied aging time. All analyses were performed using SPSS software version 24.0 (SPSS Inc., Chicago, IL, USA).

## 5. Conclusions

This study provides information on the impact of the addition of wood chips from oak and cherry during alcoholic fermentation and the maturation process on the evolution of different phenolic parameters, individual phenolic compounds, and the sensory profile of rosé wines.

The results obtained in this experimental work demonstrate that the use of wood chips during alcoholic fermentation generally induced a tendency for an increment of phenolic content and polymeric pigments in rosé musts. On the other hand, during wine maturation, rosé wines previously produced in contact with wood chips during alcoholic fermentation, as well as those that maintained this contact during maturation, showed a tendency to maintain higher values of colored anthocyanins, contributing to significantly higher color intensity values compared to control rosé wine. The positive impact of the use of wood chips was also confirmed by the significantly lower values of color hue detected during the maturation process of rosé wines with wood chip contact.

Concerning the sensorial results, only for “color intensity” and “overall appreciation” descriptors, the use of wood chip species has an apparent positive impact during the maturation process of rosé wines. This trend was particularly evident for “color intensity” descriptor, with rosé wines matured in contact with wood chips showed significantly higher scores compared to the control wine. Despite all of these differences detected between rosé wines produced with and without wood chip contact, the individual impact of each wood chip species used (oak and cherry) was not evident for the general phenolic parameters or individual phenolic compounds analyzed. The only clear difference in terms of the wood chip species used was observed for (+)-catechin content. In this case, rosé wines produced in contact with cherry chips showed the highest values.

The outcomes of our study would be of practical interest to winemakers, especially when the option for production and maturation of rosé wines through the addition of wood chips may be an option to produce wines with potential new profiles. However, further research will be necessary, especially with the introduction of other variables, such as the use of an extended aging time, other wood chip species and concentrations, and also the possibility to analyze the impact of the use of different grape varieties in producing rosé wines.

## Figures and Tables

**Table 1:** Evolution of general phenolic composition and color parameters during the alcoholic fermentation of rosé musts in contact with oak and cherry wood chips.

	Alcoholic Fermentation Days																	
	0			2			6			8			10			20		
Parameters	Rosé Musts																	
	CTM	OK	CH	CTM	OK	CH	CTM	OK	CH	CTM	OK	CH	CTM	OK	CH	CTM	OK	CH
Total phenols index (absorbance units)	10.33 ^a (1)^ ± 0.15	11.26^b^ ± 0.02	12.06^c^ ± 0.16	8.83 ^a^ ± 0.02	8.76 ^a^ ± 0.01	9.48 ^b^ ± 0.05	9.38 ^b^ ± 0.12	8.90 ^a^ ± 0.08	10.08^c^ ± 0.07	9.58 ^a^ ± 0.07	10.00 ^b^ ± 0.05	10.05 ^b^ ± 0.08	9.65 ^a^ ± 0.13	10.26 ^b^ ± 0.02	10.46 ^b^ ± 0.05	9.21 ^a^ ± 0.12	10.41 ^b^ ± 0.10	10.80^c^ ± 0.17
Non-flavonoid phenols (absorbance units)	4.06 ^a^ ± 0.12	4.98 ^b^ ± 0.11	4.79 ^b^ ± 0.01	3.88 ^b^ ± 0.05	3.69 ^a^ ± 0.03	3.75 ^a^ ± 0.03	4.00 ^a^ ± 0.15	4.11 ^a^ ± 0.02	4.55 ^b^ ± 0.01	3.90 ^a^ ± 0.19	4.41 ^a^ ± 0.06	4.23 ^a^ ± 0.33	3.92 ^a^ ± 0.03	3.89 ^a^ ± 0.05	4.17 ^b^ ± 0.02	3.62 ^a^ ± 0.02	3.85^b^ ± 0.05	4.02^c^ ± 0.02
Flavonoid phenols (absorbance units)	6.26 ^a^ ± 0.27	6.28 ^a^ ± 0.12	7.27 ^b^ ± 0.15	4.95 ^a^ ± 0.03	5.07 ^b^ ± 0.01	5.72^c^ ± 0.03	5.37 ^b^ ± 0.06	4.78 ^a^ ± 0.06	5.53 ^b^ ± 0.08	5.68 ^a^ ± 0.11	5.58 ^a^ ± 0.09	5.81 ^a^ ± 0.24	5.72 ^a^ ± 0.09	6.37 ^b^ ± 0.07	6.29 ^b^ ± 0.05	5.59 ^a^ ± 0.14	6.56 ^b^ ± 0.15	6.77 ^b^ ± 0.19
Total anthocyanins (mg/L) ^(2)^	50.78 ^a^ ± 2.02	56.92^b^ ± 4.36	67.53^c^ ± 1.23	48.12 ^b^ ± 1.04	36.88 ^a^ ± 1.38	44.78 ^b^ ± 4.25	44.14 ^b^ ± 1.15	30.74 ^a^ ± 1.11	37.76^c^ ± 3.31	60.88 ^b^ ± 1.26	29.74 ^a^ ± 1.11	30.36 ^a^ ± 3.66	46.47 ^b^ ± 2.20	24.07 ^a^ ± 1.16	27.72 ^a^ ± 2.53	42.02^c^ ± 1.14	35.04 ^b^ ± 2.34	24.04 ^a^ ± 1.09
Colored anthocyanins (mg/L) ^(2)^	14.04 ^b^ ± 0.07	9.45 ^a^ ± 0.08	9.68 ^a^ ± 0.19	11.63 ^a^ ± 0.09	13.92 ^b^ ± 0.10	14.90^c^ ± 0.04	8.37 ^a^ ± 0.03	8.97 ^a^ ± 0.01	10.29 ^b^ ± 0.03	11.31 ^b^ ± 0.02	9.38 ^a^ ± 0.06	9.23 ^a^ ± 0.08	7.88 ^b^ ± 0.03	8.23 ^c^ ± 0.03	7.42 ^a^ ± 0.10	9.17^c^ ± 0.11	8.03 ^b^ ± 0.02	7.58 ^a^ ± 0.01
Ionization degree of anthocyanins (%)	27.67 ^b^ ± 1.09	16.66^a^ ±1.16	14.33 ^a^ ± 0.34	24.18 ^a^ ± 0.48	37.77 ^b^ ± 1.16	33.48 ^b^ ± 3.22	18.97 ^a^ ± 0.47	29.21 ^a^ ± 1.10	29.28 ^a^ ± 2.64	18.58 ^a^ ± 0.34	31.56 ^b^ ± 1.38	30.67 ^b^ ± 3.27	16.99 ^a^ ± 0.89	34.25 ^b^ ± 1.73	26.90 ^c^ ± 2.33	21.84 ^a^ ± 0.72	22.98 ^a^ ± 1.51	31.56 ^b^ ± 1.48
Total pigments (absorbance units)	2.72 ^a^ ± 0.10	2.99 ^a^ ± 0.21	3.50 ^b^ ± 0.05	2.59 ^b^ ± 0.05	1.98 ^a^ ± 0.04	2.35 ^b^ ± 0.21	2.35 ^a^ ± 0.05	1.68 ^a^ ± 0.05	2.05 ^a^ ± 0.47	3.19 ^b^ ± 0.05	1.65 ^a^ ± 0.04	1.71 ^a^ ± 0.17	2.45 ^b^ ± 0.11	1.38 ^a^ ± 0.05	1.58 ^a^ ± 0.10	2.35^c^ ± 0.05	1.98 ^b^ ± 0.11	1.48 ^a^ ± 0.03
Polymeric pigments (absorbance units)	0.11^c^ ± 0.01	0.09 ^b^ ± 0.01	0.07 ^a^ ± 0.00	0.11 ^b^ ± 0.01	0.08 ^a^ ± 0.00	0.07 ^a^ ± 0.01	0.09 ^a^ ± 0.01	0.08 ^a^ ± 0.01	0.09 ^a^ ± 0.00	0.09 ^a^ ± 0.00	0.09 ^a^ ± 0.01	0.11 ^b^ ± 0.01	0.08 ^a^ ± 0.01	0.10 ^b^ ± 0.00	0.11 ^b^ ± 0.01	0.16 ^b^ ± 0.01	0.14 ^a^ ± 0.02	0.16 ^a^ ± 0.01
Polymerization degree of pigments (%)	4.13^c^ ± 0.21	3.02 ^b^ ± 0.36	2.13 ^a^ ± 0.10	4.30 ^b^ ± 0.60	4.30 ^b^ ± 0.46	3.00 ^a^ ± 0.34	3.80 ^a^ ± 0.13	5.21 ^a^ ± 2.10	5.10 ^a^ ± 1.23	2.89 ^a^ ± 0.15	5.90 ^b^ ± 0.12	7.01 ^b^ ± 1.05	3.26 ^a^ ± 0.09	7.68 ^b^ ± 0.99	7.48 ^b^ ± 0.34	6.50 ^a^ ± 0.14	7.08 ^a^ ± 0.42	11.30 ^b^ ± 0.31
Color intensity (absorbance units)	1.29 ^b^ ± 0.02	0.92 ^a^ ± 0.01	0.87 ^a^ ± 0.03	1.07 ^a^ ± 0.03	1.17 ^b^ ± 0.00	1.20 ^b^ ± 0.00	0.77 ^a^ ± 0.01	0.83 ^b^ ± 0.00	0.95^c^ ± 0.01	1.01^c^ ± 0.00	0.87 ^a^ ± 0.01	0.93 ^b^ ± 0.01	0.72 ^a^ ± 0.01	0.82 ^b^ ± 0.01	0.81 ^b^ ± 0.02	1.01^c^ ± 0.01	0.87 ^a^ ± 0.01	0.90 ^b^ ± 0.00
Color hue (absorbance units)	0.46 ^a^ ± 0.03	0.51^c^ ± 0.06	0.48 ^b^ ± 0.01	0.45^c^ ± 0.02	0.42 ^b^ ± 0.02	0.40 ^a^ ± 0.01	0.45 ^a^ ± 0.01	0.47 ^b^ ± 0.02	0.46^a, b^ ± 0.00	0.45 ^a^ ± 0.01	0.47 ^b^ ± 0.02	0.50^c^ ± 0.01	0.47 ^a^ ± 0.02	0.51 ^b^ ± 0.03	0.54 ^c^ ± 0.01	0.50 ^a^ ± 0.01	0.52 ^b^ ± 0.01	0.54 ^c^ ± 0.02
Color due to copigmentation (%)	64.38 ^a^ ± 3.11	61.19^a^ ± 0.05	63.37 ^a^ ± 6.28	68.79 ^b^ ± 2.10	44.38 ^a^ ± 10.85	63.33 ^b^ ± 5.31	45.14^a, b^ ± 2.71	39.84 ^a^ ± 5.29	53.26 ^b^ ± 1.88	63.47 ^a^ ± 13.82	44.02 ^a^ ± 0.36	48.98 ^a^ ± 3.45	43.03 ^a^ ± 2.73	41.05 ^a^ ± 0.06	48.55 ^b^ ± 0.183	61.44 ^c^ ± 2.07	32.63 ^a^ ± 1.66	43.73 ^b^ ± 6.64

Note: ^(1)^ mean values with the same letters for each parameter and for the same alcoholic fermentation day are not significantly different (Tukey’s test, *p* < 0.05); average values of three replicates; ^(2)^ expressed in malvidin-3-*O*-glucoside equivalents. CTM—rosé must fermented without wood chip contact; OK— rosé must fermented with oak wood chip contact; CH— rosé must fermented with cherry wood chip contact. Alcoholic fermentation periods: 0, 2, 6, 8, 10, and 20 days.

**Table 2:** Evolution of general phenolic composition and color parameters of rosé wines maturated in contact with oak and cherry wood chips during 80 days.

Maturation Days															
	40					60					80				
Rosé Wines															
Parameters	CTW	OKF	OKFM	CHF	CHFM	CTW	OKF	OKFM	CHF	CHFM	CTW	OKF	OKFM	CHF	CHFM
Total phenols index (absorbance units)	11.26^b(1)^ ± 0.56	10.00 ^a^ ± 0.00	10.00 ^a^ ± 0.00	11.86 ^b^ ± 0.23	11.73 ^b^ ± 0.15	10.66 ^a^ ± 0.40	9.90 ^a^ ± 0.10	10.06 ^a^ ± 0.05	11.60^b^ ± 0.17	12.06 ^b^ ± 0.55	10.76^a,b^ ± 0.58	10.06^a^ ± 0.05	10.16 ^a^ ± 0.00	11.93^c^ ± 0.58	11.70^b,c^ ± 0.17
Non-flavonoid phenols (absorbance units)	3.71 ^b^ ± 0.02	3.63 ^a^ ± 0.03	3.64 ^a^ ± 0.02	3.66^a, b^ ± 0.00	3.82^c^ ± 0.04	3.70 ^b^ ± 0.00	3.58^c^ ± 0.06	3.65 ^b^ ± 0.01	3.67^a, b^ ± 0.01	3.78 ^a^ ± 0.01	3.81^c^ ± 0.00	3.59 ^a^ ± 0.02	3.63 ^a^ ± 0.01	3.73 ^b^ ± 0.02	3.78^c^ ± 0.01
Flavonoid phenols (absorbance units)	7.55 ^b^ ± 0.54	6.37 ^a^ ± 0.03	6.36 ^a^ ± 0.02	8.20 ^b^ ± 0.22	7.91 ^b^ ± 0.11	6.96 ^a^ ± 0.40	6.31 ^a^ ± 0.14	6.41 ^a^ ± 0.04	7.92 ^b^ ± 0.16	8.28 ^b^ ± 0.55	6.95^a, b^ ± 0.58	6.47 ^a^ ± 0.03	6.53 ^a^ ± 0.05	8.20^c^ ± 0.57	7.91 ^b,c^ ± 0.15
Total anthocyanins (mg/L) ^(2)^	36.40^b^ ± 3.46	31.57^a,b^ ± 1.15	31.62^a,b^ ± 1.11	31.64^a,b^ ± 1.88	24.93 ^a^ ± 5.99	32.51 ^a^ ± 2.67	33.24 ^a^ ± 1.09	31.14 ^a^ ± 1.13	28.36^a^ ±0.07	31.38 ^a^ ± 3.19	32.94 ^a^ ± 1.41	34.09^a^ ± 1.30	29.58 ^a^ ± 4.19	33.55^a^ ± 4.02	32.87 ^a^ ± 1.95
Colored anthocyanins (mg/L) ^(2)^	2.10 ^a^ ± 0.02	5.02 ^b^ ± 0.02	5.31^c^ ± 0.02	4.96 ^b^ ± 0.20	5.08 ^b,c^ ± 0.03	2.14 ^a^ ± 0.06	4.04^c^ ± 0.01	5.06 ^b^ ± 0.17	4.29^d^ ± 0.04	4.59 ^b^ ± 0.07	1.72 ^a^ ± 0.08	3.28^d^ ± 0.02	4.07 ^b^ ± 0.02	3.39^c^ ± 0.04	4.36 ^b^ ± 0.03
Ionization degree of anthocyanins (%)	5.80 ^a^ ± 0.51	15.91 ^b^ ± 0.59	16.81 ^b^ ± 0.56	15.73 ^b^ ± 1.55	21.26 ^b^ ± 5.58	6.64 ^a^ ± 0.78	12.18 ^b^ ± 0.36	16.25^c^ ± 0.50	15.13^c^ ± 0.14	14.72^c^ ± 1.26	5.25 ^a^ ± 0.48	9.64 ^b^ ± 0.30	13.96^c^ ± 2.06	10.21^b^ ± 1.22	13.31^c^ ± 0.70
Total pigments (absorbance units)	1.95 ^b^ ± 0.15	1.78^a, b^ ± 0.05	1.78^a, b^ ± 0.05	1.81^a, b^ ± 0.10	1.44 ^a^ ± 0.30	1.75 ^a^ ± 0.11	1.85 ^a^ ± 0.05	1.75 ^a^ ± 0.05	1.61 ^a^ ± 0.01	1.75 ^a^ ± 0.15	1.78 ^a^ ± 0.05	1.88 ^a^ ± 0.05	1.65 ^a^ ± 0.21	1.88 ^a^ ± 0.21	1.81 ^a^ ± 0.10
Polymeric pigments (absorbance units)	0.07 ^a^ ± 0.01	0.12 ^b^ ± 0.01	0.12 ^b^ ± 0.01	0.15^c^ ± 0.01	0.12 ^b^ ± 0.02	0.07 ^a^ ± 0.01	0.11 ^b^ ± 0.01	0.11 ^b^ ± 0.01	0.11 ^b^ ± 0.01	0.10 ^b^ ± 0.01	0.08 ^a^ ± 0.01	0.10 ^b^ ± 0.01	0.10 ^b^ ± 0.01	0.13^c^ ± 0.00	0.10 ^b^ ± 0.01
Polymerization degree of pigments (%)	4.11 ^a^ ± 0.94	6.91 ^b^ ± 0.22	6.84 ^b^ ± 0.16	7.78 ^b^ ± 0.24	8.56 ^b^ ± 1.59	4.32 ^a^ ± 0.92	6.14 ^b^ ± 0.08	6.63 ^b^ ± 0.17	7.34 ^b^ ± 0.14	6.26 ^b^ ± 0.89	4.62 ^a^ ± 0.58	5.75^a,b^ ± 0.40	6.27 ^b^ ± 0.82	6.64 ^b^ ± 0.43	5.76^a, b^ ± 0.25
Color intensity (absorbance units)	0.35 ^a^ ± 0.03	0.63 ^b^ ± 0.00	0.65 ^b^ ± 0.00	0.68 ^b^ ± 0.01	0.64 ^b^ ± 0.01	0.34 ^a^ ± 0.04	0.54 ^b^ ± 0.01	0.62^c^ ± 0.01	0.58 ^b,c^ ± 0.01	0.58 ^b,c^ ± 0.02	0.35 ^a^ ± 0.02	0.48 ^b^ ± 0.01	0.52 ^b,c^ ± 0.00	0.54^c^ ± 0.01	0.55^c^ ± 0.01
Color hue (absorbance units)	0.81^c^ ± 0.02	0.62 ^a, b^ ± 0.01	0.61 ^a^ ± 0.01	0.66 ^b^ ± 0.00	0.63^a, b^ ± 0.01	0.79 ^b^ ± 0.04	0.65 ^a^ ± 0.02	0.61 ^a^ ± 0.01	0.66 ^a^ ± 0.02	0.63 ^a^ ± 0.01	0.93^d^ ± 0.03	0.68 ^b^ ± 0.01	0.63 ^a^ ± 0.01	0.73^c^ ± 0.01	0.63 ^a^ ± 0.01
Color due to copigmentation (%)	41.48 ^a^ ± 2.90	36.83 ^a^ ± 0.16	36.64 ^a^ ± 2.01	35.50 ^a^ ± 2.34	41.95 ^a^ ± 6.58	43.76 ^b^ ± 0.13	38.65^a,b^ ± 0.23	37.46^a,b^ ± 2.35	36.05^a^ ± 5.62	41.53^a,b^ ± 0.45	43.02 ^a^ ± 0.61	38.76^a^ ± 2.48	42.90 ^a^ ± 2.59	38.75^a^ ± 6.28	42.40 ^a^ ± 0.35

Note: ^(1)^ mean values with the same letters for each parameter and for the same maturation day are not significantly different (Tukey’s test, *p* < 0.0.5); average values of three replicates; ^(2)^ expressed in malvidin-3-*O*-glucoside equivalents. CTW—control wine; OKF—wine produced with oak wood chip contact only during alcoholic fermentation; OKFM—wine produced with oak wood chip contact during alcoholic fermentation and maturation process; CHF—wine produced with cherry wood chip contact only during alcoholic fermentation; CHFM—wine produced with cherry wood chip contact during alcoholic fermentation and maturation process. Maturation periods: 40, 60, and 80 days.

**Table 3:** Experimental conditions and corresponding rosé must and wine codes used in this study.

Experimental Conditions	Sample Codes
Alcoholic fermentation	
Rosé must without wood chip contact	CTM
Rosé must with oak (*Quercus petraea*) wood chip contact	OK
Rosé must with cherry (*Prunus avium*) wood chip contact	CH
Aging process	
Rosé wine produced without wood chip contact during alcoholic fermentation and maturation process	CTW
Rosé wine produced with oak (*Quercus petraea*) wood chip contact only during alcoholic fermentation	OKF
Rosé wine produced with oak (*Quercus petraea*) wood chip contact during alcoholic fermentation and maturation process	OKFM
Rosé wine produced with cherry (*Prunus avium*) wood chip contact only during alcoholic fermentation	CHF
Rosé wine produced with cherry (*Prunus avium*) wood chip contact during alcoholic fermentation and maturation process	CHFM
